# Trends of Adherence to the Mediterranean Dietary Pattern in Northern Italy from 2010 to 2016

**DOI:** 10.3390/nu9070734

**Published:** 2017-07-11

**Authors:** Alessandro Leone, Alberto Battezzati, Ramona De Amicis, Giulia De Carlo, Simona Bertoli

**Affiliations:** International Center for the Assessment of Nutritional Status (ICANS), Department of Food, Environmental and Nutritional Sciences (DeFENS), University of Milan, Via Sandro Botticelli 21, 20133 Milan, Italy; alberto.battezzati@unimi.it (A.B.); ramona.deamicis@unimi.it (R.D.A.); giulia.decarlo@unimi.it (G.D.C.); simona.bertoli@unimi.it (S.B.)

**Keywords:** Mediterranean diet, determinants, prevalence, trends, food consumption, Italy

## Abstract

Little information is available on the trends of adherence to the Mediterranean dietary pattern (MDP). This study investigates food consumption trends from 2010 to 2016 in subjects living in Northern Italy. A cross-sectional study of 8584 subjects enrolled between January 2010 and December 2016 was conducted. Socio-demographic, nutrition and lifestyle characteristics were collected. A 14-item questionnaire was used to evaluate adherence to MDP. Multivariable Poisson regression was used to evaluate the trends of and the determinants for the adherence to MDP. The overall prevalence of adherence to MDP was 14% and the trend remained constant over the six years. However, there was a marked increase in nuts consumption and a slight one in white meat consumption. Furthermore, we observed a decrease in the consumption of fruit, red meat, sweets and sugar-sweetened beverages and in the use of soffritto. Finally, higher education, being older, married, physically active, and ex-smoker was associated with greater adherence to MDP, whereas the prevalence of adherence was lower in the obese. In conclusion, the consumption of some Mediterranean and non-Mediterranean food groups changed over the six years. However, overall, the prevalence of adherence to MDP did not change. Additional strategies promoting healthy dietary habits are needed.

## 1. Introduction

The Mediterranean diet was defined as the traditional dietary pattern of people living in Greece, Southern Italy, Spain and other countries of the Mediterranean basin [1]. It is characterized by a high consumption of cereals, vegetables, fruits, nuts, legumes, seeds and olives, with olive oil as principal source of added fat, along with high-to-moderate intakes of fish and seafood, moderate consumption of eggs, poultry and dairy products (cheese and yogurt), low consumption of red meat and a moderate intake of alcohol (mainly wine during meals) [1]. In 2010, this dietary pattern has been recognized as an Intangible Cultural Heritage of Humanity by United Nations Educational, Scientific and Cultural Organization (UNESCO).

After the Seven Countries Study [2], which observed low death rates for all-cause and coronary heart disease in cohorts where olive oil was used as the main fat, numerous and increasing epidemiological studies and trials have established the health benefits associated with adherence to the Mediterranean dietary pattern (MDP), mainly in relation to reducing the risk of developing CVD [3], metabolic syndrome [4], type 2 diabetes [5], cancer [6] and some neurodegenerative diseases [7]. More recently, several prospective and cross-sectional studies have observed an inverse association between the adherence to the MDP and the risk of mental diseases like depression [8] and eating disorders [9].

Despite these documented health benefits, there has been a rapid decline in MDP adherence in recent decades [10]. Indeed, an early retrospective study noted this, especially in Mediterranean populations, on comparing MDP adherence in the 2000–2003 period to that of 1961–1965 [11]. Subsequently, from 2005 to 2010, a deep decline in adherence to MDP was recorded in the population of Southern Italy, with prevalence falling from over 30% to 18% [12]. A study conducted in Spain in 2008–2010 recorded that only 12% of subjects had dietary habits in accordance with MDP [13], while a previous study in Northern Italy reported constant adherence to the MDP during the 1991–2006 period [14]. However, in this latter study, no information was given about the prevalence of adherence to MDP. Moreover, since 2010, no study has investigated the trends of adherence to MDP. Therefore, the aim of the present study was to investigate the trends of adherence to MDP from 2010 to 2016 in a large cohort of subjects living in Northern Italy.

## 2. Materials and Methods 

### 2.1. Study Design

We performed a cross-sectional study on 8584 consecutive adults who self-referred to the International Center for the Assessment of Nutritional Status (ICANS, University of Milan) from January 2010 to December 2016, in order to participate in a structured weight loss or weight maintenance program. On the same day, they underwent a clinical examination, an anthropometric assessment and a structured interview by trained dieticians to obtain information about marital status, educational level, smoking and structured physical activity. Subjects who spent ≥2 h per week in any structured physical activity were considered as active [15]. All the patients filled in a questionnaire to evaluate MDP adherence [16]. Exclusion criteria were: age < 18, subjects undergoing medical nutritional therapy for any condition or following any kind of weight loss dietary regimen in the last six months, subjects with known problem of alcohol abuse, subjects diagnosed as having acute infective, neurological, gastrointestinal, cardiac, renal and pulmonary failure, or who were unable to understand and fill in the questionnaires. This study was conducted according to the guidelines laid down in the Declaration of Helsinki and all procedures involving human subjects were approved by the Ethics Committee of the University of Milan (23/16). Written informed consent was obtained from all subjects.

### 2.2. Anthropometric Measurements

Anthropometric measurements were made according to the conventional criteria and measuring procedures proposed by Lohmann et al. [17]. Weight, to 100 g, was measured on a Column scale (Seca 700 balance, Seca Corporation, Hanover, MD, USA) with subjects wearing only light underwear and an empty bladder. Height was measured to the nearest 0.1 cm using a vertical stadiometer (Seca 217 stable stadiometer, Seca Corporation, Hanover, MD, USA). Waist circumference (WC) was measured midway between the lower rib margin and the superior anterior iliac spine taken to the nearest 0.5 cm, and measured with a non-stretch tape applied horizontally.

### 2.3. Adherence to the Mediterranean Dietary Pattern

Adherence to MDP was evaluated using a validated 14-item questionnaire [16]. Schröder et al. [16] found a good correlation (*r* = 0.52) and a good absolute agreement (intraclass correlation coefficient (ICC) = 0.51) between the 14-item Mediterranean score (MedScore) and the Mediterranean score obtained from a 136-item food frequency questionnaire, concluding that this short screener was a valid tool for a rapid assessment of adherence to MDP. The MedScore was obtained from this questionnaire following the guidelines of the Prevención con Dieta Mediterránea (PREDIMED) study group (www.predimed.es) with some adaptation already used in previous studies [9,18,19,20]. Briefly, one point was attributed for each of the following: (1) olive oil as the main cooking fat; (2) olive oil ≥ 4 tablespoons/day; (3) vegetables ≥ 2 servings/day (≥1 portion raw or on salad); (4) fruit ≥ 3 servings/day; (5) red or processed meat < 1 serving/day; (6) butter or cream or margarine < 1/day; (7) sugar-sweetened beverages < 1/day; (8) wine ≥ 3 glasses/week; (9) legumes ≥ 3 servings/week; (10) fish/seafood ≥ 3 servings/week; (11) commercial sweets and confectionery < 3/week; (12) nuts ≥ 1/week; (13) white more than red meats (yes) and; (14) use of soffritto ≥ 2/week. Subjects with a MedScore ≥ 9 points were considered to have a dietary pattern in accordance with the MDP [9,18,19,20]. 

### 2.4. Statistical Analysis

Most continuous variables had non-Gaussian distributions, and all are reported as 25th, 50th and 75th percentiles. Discrete variables are reported as counts and percentages. A Poisson working regression model (PWRM) with a robust 95% confidence interval was used to estimate prevalence and prevalence ratios (PR) of adherence to MDP and to its individual components. Uni- and multivariable PWRM were used to evaluate the association of adherence to MDP, and individual components, with sex, age, body mass index (BMI), educational level, occupation, marital status, smoking status, physical activity and year of recruitment. The outcome variables of all models (adherence to MDP and to its individual components) were discrete (0 = no; 1 = yes). The covariates were coded as follows: (i) sex (discrete, 0 = female; 1 = male); (ii) age (continuous, years); (iii) BMI (continuous, kg/m^2^); (iv) educational level (discrete, 0 = ≤high school; 1 = >high school); (v) occupation (discrete, 0 = unemployed/student/retired/housewife, 1 = worker); (vi) marital status (discrete, 0 = single/widower; 1 = married/cohabiting); (vii) smoking status (discrete, 0 = non-smoker; 1 = smoker; 2 = ex-smoker); (viii) physical activity (discrete, 0 = no; 1 = yes) and (ix) year of recruitment (ordinal, 0 = 2010; 1 = 2011; 2 = 2012; 3 = 2013; 4 = 2014; 5 = 2015; 6 = 2016). Prevalence ratios and marginal probabilities were calculated from PWRM [21]. Tests of linear trend across increasing year of recruitment were conducted treating the variable as a continuous variable. Uni- and multivariable fractional polynomials (MFP) were used to test whether the relationships of continuous predictors with the outcomes were non-linear [22,23]. Using this approach, no transformation or different transformations of age and BMI were selected in function of the outcome of interest. However, as there was only a modest gain in the linearity of continuous predictors when MFP were applied to the PWRM, all continuous covariates were kept untransformed with the benefit of making the relationships more understandable to a clinical audience [15]. Statistical analysis was performed using Stata 12.1 (Stata Corporation, College Station, TX, USA). 

## 3. Results

Overall characteristics of the recruited sample are reported in Table 1.

Socio-anagraphic, nutritional status and lifestyle characteristics of the sample are reported in Table 2.

In the pool sample, 14% of subjects had a dietary pattern in accordance with MDP. Table 3 shows the trends of and the determinants for the adherence to MDP and individual components expressed as PRs, and the Supplementary Table S1 shows the corresponding predicted probabilities.

We observed an increment in the consumption of nuts (*p* for trend < 0.001) and white meat (*p* for trend < 0.001) and a decrement in the consumption of fruit (*p* for trend < 0.001), red meat (*p* for trend = 0.005), sweets (*p* for trend = 0.026), sugar-sweetened beverages (*p* for trend < 0.001) and use of soffritto (*p* for trend < 0.001). The consumption of olive oil was higher during the 2011–2016 period, compared to 2010, but the linear trend was not significant (*p* for trend = 0.565). The adherence to MDP did not change during the period of interest.

With regard to factors associated with the adherence to the MDP, we found that age (PR = 1.03, 95% CI: 1.03–1.04; for each 1-year increase), having a higher education (PR = 1.13, 95% CI: 1.01–1.26), being married (PR = 1.15, 95% CI: 1.03–1.28), ex-smoker (PR = 1.22, 95% CI: 1.08–1.38) and physically active (PR = 1.37, 95% CI: 1.23–1.52) were directly associated with the prevalence of adherence to the MDP. On the contrary, BMI (PR = 0.98, 95% CI: 0.97–0.99; for each 1-kg/m^2^ increase) was inversely associated with the prevalence of adherence to the MDP. Sex and having a job were not associated with the prevalence of adherence to MDP, although this latter was almost significant. 

Supplementary Figure S1 plots the prevalence of adherence to MDP and individual components estimated from the PWRM as a function of age and BMI. It can be seen that the adherence to MDP increased with increasing age and decreased with increasing BMI levels.

## 4. Discussion

In this study, we evaluated the trends of adherence to MDP and the consumption of individual food groups during the period 2010–2016. We found that in Northern Italy, only 14% of the subjects had a dietary pattern consistent with MDP, and this did not change over the six years. However, the consumption of some Mediterranean and non-Mediterranean food groups changed during these years. We observed a marked increase in the consumption of nuts and a slight one of white meat consumption. On the other hand, there was a decrease in the consumption of fruit, sweets and sugar-sweetened beverages and in the use of soffritto. Moreover, we found that age, nutritional status, having higher education and being married, an ex-smoker and physically active were factors associated with better adherence to MDP. 

Despite its health benefits, adherence to MDP has declined in the last decades. Indeed, da Silva et al. [11] found a decrease in the level of adherence to MDP in the period 2000–2003 compared to 1961–1965. This was more evident among subjects of Mediterranean countries. Bonaccio at el. [12] recorded a deep decline in adherence to MDP, the prevalence falling from over 30% to 18% among subjects included in the Molisani study during the period 2005–2010. Ecological studies have reported a substantial departure from MDP all over Europe, but this is more evident in Mediterranean countries that have experienced a “westernisation” process of food habits [24]. Furthermore, the increasing cost of many key-foods of the Mediterranean diet has been proposed as a factor, driving people to give up this eating pattern in favour of less expensive, energy-dense foods that typically have lower nutritional quality [25]. Our study, started in 2010, shows that the food pattern of only 14% of individuals was in accordance with MDP, and the trend remained constant over the six years. The prevalence of adherence was slightly lower than that recorded in 2010 in Southern Italy [12], and slightly higher than the prevalence of adherence reported in Spain in 2008–2010. However, despite these little discrepancies between regions and countries, presumably because of the different food cultures, and the different index used for defining MDP, the overall prevalence was low. This highlights the importance of outreach interventions that promote the health benefits of following a dietary pattern consistent with MDP. 

Our study also shows the trends of consumption of individual Mediterranean and non-Mediterranean food groups. Indeed, even though adherence to MDP did not change over the six years, the consumption of some foods changed. Indeed, we recorded a strong increase in nuts consumption, and hypothesize that this was due to the dissemination of results of recent trials and observational cohorts suggesting a lower risk of disease associated with the consumption of nuts [26]. In addition to nuts consumption, we also observed a slight, but positive increase in the consumption of white meat with a concomitant decrease in that of red meat. This could be a consequence of both the economic crisis, which has led people to prefer, for price reasons, white meat rather than red, and of the World Health Organization (WHO) recommendation to reduce the consumption of meat products. Also positive is the slight decrease recorded for the consumption of sugar-sweetened beverages and sweets. However, we also registered a decrease in the consumption of fruit and the use of soffritto, this latter presumably because of the little time dedicated to the food preparation. 

Consistently with some [27], but not all [12,13,28], previous findings, we did not find any sex difference in MDP adherence. A possible explanation could be that, differently from the past, men now tend to participate in food purchasing and meal preparation.

With regard to age, the older subjects reported a higher consumption of Mediterranean food, tending to avoid non-Mediterranean foods, while the younger ones did the opposite, the only exception being their use of olive oil and soffritto. Such dietary habits led to the older groups having a higher adherence to MDP than the younger. The prevalence values ranged from nearly 0% for the younger subjects to around 30–40% for the older. It is possible that the older subjects simply maintained traditional dietary habits acquired in infancy, thus remaining less affected by the process of diet-westernization, while young people tend to be more willing to accept food from other cultures. Therefore, our results support the hypothesis of a departure, also in Northern Italy, from the MDP. 

Concerning nutritional status, adherence to MDP decreased with increasing BMI. Indeed, lower BMI was associated with greater wine and nuts consumption, whereas a higher BMI was associated with a greater consumption of red meat and sugar-sweetened beverages, and also of the use of olive oil and soffritto. The inverse association between adherence to the Mediterranean diet and obesity has been reported by several cross-sectional [18] and longitudinal studies [29]. Similarly, a high consumption of meat and processed meat [30], as well as sugar-sweetened beverages [31], has been associated with an increased risk of obesity. The thorniest association is that of olive oil and risk of obesity. Observational and intervention trials have consistently shown that a Mediterranean diet rich in olive oil does not contribute to obesity, and may actually help curb it [32]. Indeed, a PREDIMED study showed that an olive oil-rich diet was effective in the prevention of weight gain [33]. Independently of the Mediterranean diet, few data are available on the role of olive oil in preventing or managing obesity [32]. In a previous cross-sectional study, we found that olive oil consumption was associated with a higher prevalence of obesity and greater abdominal visceral adipose thickness [18]. In fact, the obese subjects in our study had a lower MDP adherence, therefore, it is possible that high olive oil consumption associated with dietary habits not representative of MDP could increase the risk of developing obesity.

Our results suggest that being married, or cohabiting, increases MDP adherence, a result that agrees with previous investigations [28]. It also agrees with the recommendation that people eat together around a table, as sharing food in the company of family and friends represents social support and gives a sense of community; furthermore, the pleasure associated with the conviviality of meals can affect food behaviour positively, and in turn, health status [34]. 

Our study has shown that adherence to MDP increased with educational level, a finding consistent with previous investigations [13]. In fact, it is quite plausible that those who have a higher educational level are more aware of the role of nutrition on health status. 

Consistently with previous studies, the ex-smokers had a higher adherence to MDP [13,28]. Indeed, ex-smokers are more likely to make positive decisions concerning their health and, as previous studies have suggested, they are more health-conscious and have a responsible profile [35]. 

Physical activity during leisure time was positively associated with adherence to MDP. These results are similar to those of previous investigations [13,28] and suggest that those who practice physical activity are more health conscious and probably more aware of the role of nutrition on health status, body composition and physical performance. 

The first strength of our study is that, to the best of our knowledge, it is the first study to investigate the trends of adherence to MDP in Northern Italy since the last study published in 2010. Moreover, the large sample size has ensured more accurate estimates, as can be seen by the restricted confidence intervals. In addition, we used a validated questionnaire, previously used in the PREDIMED trial, for defining the accordance to MDP. However, we could not evaluate the individual amounts of each food or the macro and micro-nutrient intake, the first limitation of our study. The second is that we used a cross-sectional design, which did not allow us to evaluate the intra-person changes in food consumption and the cause–effect relationship. Moreover, the recruitment of different people every year can theoretically be a further explanation of our results. However, we observed similar socio-demographic, nutritional and lifestyle characteristics of subjects according to year of recruitment, and, therefore, we do not have reason to think that the recruitment has affected our findings. The third is that the enrolled subjects were seeking weight loss or a maintenance programme, which might affect the general applicability of our findings to other groups or populations. The fourth limitation is that, although in this study, during the time-period of six years, we have found some increments or decrements in the consumption of several foods, we cannot rule out that some dietary habits can take longer to be modified. Therefore, the long-term evaluation of the food consumption trends is needed to confirm these results. The fifth is the lack of model adjustment for individual income; however, we were able to adjust for education and occupation, thus reflecting the socioeconomic status of the recruited subjects. The sixth is that we considered only leisure physical activity, without considering the physical activity at work. Finally, as in any observational study, potential residual confounding cannot be ruled out.

## 5. Conclusions

In conclusion, food consumption is in constant flux, undergoing continuous and constant mutation, presumably in response to globalization processes, economic conditions, and also to the dissemination of scientific research results. Some of these changes have led to an improvement in diet quality, others to its impoverishment. So much so that, overall, the prevalence of adherence to MDP did not change over the six studied years, remaining constant low. As dietary habits have an impact on the risk of chronic diseases, these results have implications for public health, as this departure from the MDP for a more Western dietary pattern may increase the risk of disease. Therefore, further strategies are needed to promote healthy dietary habits, especially among young subjects and individuals at risk of poor diet quality and, therefore, preserving present-day MDP knowledge for younger and future generations.

## Figures and Tables

**Table 1:** General characteristics of the studied sample according to the year of recruitment.

	**2010 (*n* = 1251)**			**2011 (*n* = 1203)**			**2012 (*n* = 1212)**			**2013 (*n* = 1242)**		
	**P25**	**P50**	**P75**	**P25**	**P50**	**P75**	**P25**	**P50**	**P75**	**P25**	**P50**	**P75**
Age (years)	37	45	55	38	46	55	38	47	56	37	45	55
BMI (kg/m^2^)	25.6	28.7	32.2	25.4	28.7	32.3	25.4	28.6	32.5	25.2	28.3	32.4
WC (cm)	85.3	95.1	105.5	85.5	95.0	105.5	86.6	96.2	106.0	86.0	95.7	106.6
MedScore	5	7	8	6	7	8	5	7	8	5	7	8
	**2014 (*n* = 1192)**			**2015 (*n* = 1244)**			**2016 (*n* = 1240)**			**Total (*n* = 8584)**		
	**P25**	**P50**	**P75**	**P25**	**P50**	**P75**	**P25**	**P50**	**P75**	**P25**	**P50**	**P75**
Age (years)	38	47	55	37	46	54	37	47	55	37	46	55
BMI (kg/m^2^)	25.5	28.5	32.3	25.1	28.2	32.3	25.4	28.6	33.0	25.4	28.6	32.4
WC (cm)	87.7	97.2	106.3	86.8	96.0	106.9	88.0	98.3	108.0	86.5	96.0	106.5
MedScore	6	7	8	6	7	8	6	7	8	6	7	8

Abbreviation: P25 = 25th percentile; P50 = 50th percentile; P75 = 75th percentile; BMI = body mass index; WC = waist circumference.

**Table 2:** Socio-anagraphic, nutritional status and lifestyle characteristics of the sample according to the year of recruitment.

	2010		2011		2012		2013		2014		2015		2016		Total	
	*N*	%	*N*	%	*N*	%	*N*	%	*N*	%	*N*	%	*N*	%	*N*	%
**Sex**																
Female	877	70.1	848	70.5	862	71.1	910	73.3	846	71	880	70.7	886	71.5	6109	71.2
Male	374	29.9	355	29.5	350	28.9	332	26.7	346	29	364	29.3	354	28.5	2475	28.8
**Age Categories**																
18–19 years	16	1.3	14	1.2	17	1.4	18	1.4	14	1.2	21	1.7	20	1.6	120	1.4
20–29 years	115	9.2	106	8.8	112	9.2	123	9.9	101	8.5	110	8.8	122	9.8	789	9.2
30–39 years	280	22.4	248	20.6	224	18.5	262	21.1	232	19.5	243	19.5	234	18.9	1723	20.1
40–49 years	355	28.4	360	29.9	373	30.8	371	29.9	350	29.4	379	30.5	355	28.6	2543	29.6
50–59 years	271	21.7	269	22.4	287	23.7	266	21.4	289	24.2	307	24.7	309	24.9	1998	23.3
60–69 years	166	13.3	169	14	156	12.9	157	12.6	156	13.1	133	10.7	150	12.1	1087	12.7
≥70 years	48	3.8	37	3.1	43	3.5	45	3.6	50	4.2	51	4.1	50	4.0	324	3.8
**BMI Classes**																
Normal weight	256	20.5	268	22.3	262	21.6	291	23.4	250	21	307	24.7	271	21.9	1905	22.2
Overweight	499	39.9	469	39	471	38.9	458	36.9	477	40	462	37.1	461	37.2	3297	38.4
Obesity 1 class	342	27.3	306	25.4	304	25.1	329	26.5	311	26.1	309	24.8	305	24.6	2206	25.7
Obesity 2 and 3 class	154	12.3	160	13.3	175	14.4	164	13.2	154	12.9	166	13.3	203	16.4	1176	13.7
**Education**																
Low degree	757	60.5	787	65.4	758	62.5	727	58.5	677	56.8	671	53.9	678	54.7	5055	58.9
High degree	494	39.5	416	34.6	454	37.5	515	41.5	515	43.2	573	46.1	562	45.3	3529	41.1
**Occupation**																
Unworker	256	20.5	280	23.3	271	22.4	295	23.8	235	19.7	242	19.5	265	21.4	1844	21.5
Worker	995	79.5	923	76.7	941	77.6	947	76.2	957	80.3	1002	80.5	975	78.6	6740	78.5
**Marital Status**																
Single	546	43.6	531	44.1	564	46.5	598	48.1	551	46.2	595	47.8	594	47.9	3979	46.4
Married	705	56.4	672	55.9	648	53.5	644	51.9	641	53.8	649	52.2	646	52.1	4605	53.6
**Smoking**																
Non-smoker	667	53.3	639	53.1	661	54.5	680	54.8	617	51.8	631	50.7	736	59.4	4631	53.9
Smoker	251	20.1	252	20.9	258	21.3	237	19.1	246	20.6	357	28.7	313	25.2	1914	22.3
Ex-smoker	333	26.6	312	25.9	293	24.2	325	26.2	329	27.6	256	20.6	191	15.4	2039	23.8
**Physical Activity**																
No	715	57.2	749	62.3	761	62.8	712	57.3	638	53.5	606	48.7	663	53.5	4844	56.4
Yes	536	42.8	454	37.7	451	37.2	530	42.7	554	46.5	638	51.3	577	46.5	3740	43.6
**Adherence to the Mediterranean Dietary Pattern**																
No	1095	87.5	1030	85.6	1040	85.8	1077	86.7	1023	85.8	1057	85.0	1062	85.6	7384	86.0
Yes	156	12.5	173	14.4	172	14.2	165	13.3	169	14.2	187	15.0	178	14.4	1200	14.0

**Table 3:** Trends of and determinants for adherence to the Mediterranean dietary pattern (MDP) and its individual food-items.

	MDP (Yes)	Olive Oil (Yes)	Olive Oil (≥4 sp/Day)	Vegetable (≥2 s/Day)	Fruits (≥3 u/Day)	Red Meat (<1 s/Day)	Animal Fats (<1 s/Day)	Sweetened Beverages (<1 gl/Day)	Wine (≥3 gl/Week)	Legumes (≥3 s/Week)	Fish (≥3 s/Week)	Sweets (<3 t/Week)	Nuts (≥1 s/Week)	White Meat (Yes)	Soffrito (≥2 t/Week)
**Year**															
2010	Ref.	Ref.	Ref.	Ref.	Ref.	Ref.	Ref.	Ref.	Ref.	Ref.	Ref.	Ref.	Ref.	Ref.	Ref.
2011	1.18	1.00	1.37 ***	1.07	0.89	1.02	1.00	1.02	1.09	1.02	0.83	1.05	1.28 *	1.01	0.95
	[0.97,1.43]	[0.99,1.02]	[1.22,1.53]	[1.00,1.15]	[0.74,1.08]	[0.96,1.08]	[0.99,1.01]	[0.99,1.06]	[0.97,1.22]	[0.72,1.44]	[0.65,1.07]	[0.97,1.13]	[1.02,1.61]	[0.94,1.08]	[0.89,1.02]
2012	1.16	1.00	1.49 ***	0.96	0.75 **	1.05	1.00	1.00	1.18 **	0.84	0.77 *	1.09 *	1.55 ***	1.03	0.66 ***
	[0.96,1.42]	[0.98,1.01]	[1.33,1.67]	[0.89,1.03]	[0.61,0.92]	[1.00,1.11]	[0.99,1.02]	[0.97,1.04]	[1.05,1.32]	[0.59,1.22]	[0.60,1.00]	[1.02,1.17]	[1.25,1.92]	[0.96,1.10]	[0.60,0.72]
2013	1.07	1.00	1.44 ***	0.98	0.81 *	1.05	1.01	1.02	1.10	0.71	0.84	1.05	1.81 ***	1.09 **	0.56 ***
	[0.87,1.30]	[0.99,1.01]	[1.28,1.61]	[0.91,1.06]	[0.67,0.98]	[1.00,1.11]	[0.99,1.02]	[0.99,1.06]	[0.98,1.24]	[0.48,1.03]	[0.66,1.07]	[0.98,1.13]	[1.47,2.23]	[1.03,1.17]	[0.51,0.62]
2014	1.10	1.00	1.19 **	1.06	0.65 ***	1.01	1.01	1.03	1.12	1.04	0.87	1.11 **	2.16 ***	1.10 **	0.59 ***
	[0.90,1.34]	[0.99,1.02]	[1.06,1.34]	[0.98,1.13]	[0.53,0.80]	[0.95,1.06]	[0.99,1.02]	[0.99,1.07]	[1.00,1.26]	[0.73,1.46]	[0.68,1.11]	[1.03,1.19]	[1.76,2.64]	[1.03,1.18]	[0.54,0.65]
2015	1.18	1.00	1.19 **	1.06	0.65 ***	1.05	1.00	1.05 **	1.09	0.90	0.82	1.09 *	2.53 ***	1.07 *	0.61 ***
	[0.97,1.44]	[0.99,1.01]	[1.06,1.34]	[0.99,1.14]	[0.53,0.80]	[1.00,1.11]	[0.99,1.02]	[1.02,1.09]	[0.97,1.22]	[0.63,1.28]	[0.64,1.04]	[1.01,1.17]	[2.08,3.08]	[1.01,1.15]	[0.55,0.66]
2016	1.16	1.01	1.18**	1.04	0.60 ***	1.09 ***	1.00	1.06 ***	1.08	0.96	0.93	1.07	2.75 ***	1.10 **	0.60 ***
	[0.95,1.41]	[0.99,1.02]	[1.04,1.33]	[0.97,1.12]	[0.49,0.74]	[1.04,1.15]	[0.99,1.02]	[1.03,1.10]	[0.96,1.22]	[0.68,1.36]	[0.73,1.18]	[1.00,1.16]	[2.27,3.34]	[1.03,1.17]	[0.55,0.66]
*P* for trend	0.292	0.455	0.761	0.142	<0.001	0.005	0.390	<0.001	0.376	0.818	0.724	0.026	<0.001	<0.001	<0.001
**Sex**															
Women	Ref.	Ref.	Ref.	Ref.	Ref.	Ref.	Ref.	Ref.	Ref.	Ref.	Ref.	Ref.	Ref.	Ref.	Ref.
Men	0.99	0.99	0.83 ***	0.84 ***	0.89	0.98	1.00	0.95 ***	1.89 ***	1.56 ***	0.82 *	1.04	0.98	0.83 ***	1.12 ***
	[0.88,1.11]	[0.99,1.00]	[0.77,0.88]	[0.80,0.88]	[0.78,1.01]	[0.95,1.01]	[0.99,1.01]	[0.93,0.97]	[1.77,2.01]	[1.27,1.92]	[0.70,0.97]	[1.00,1.09]	[0.88,1.08]	[0.80,0.87]	[1.06,1.19]
**Age** (Years)	1.03 ***	1.00 ***	1.00 *	1.01 ***	1.04 ***	1.01 ***	1.00	1.00 ***	1.02 ***	1.02 ***	1.01 ***	1.00 ***	1.03 ***	1.00 ***	1.00 *
	[1.03,1.04]	[1.00,1.00]	[0.99,1.00]	[1.01,1.01]	[1.03,1.04]	[1.01,1.01]	[1.00,1.00]	[1.00,1.00]	[1.02,1.02]	[1.01,1.02]	[1.01,1.02]	[1.00,1.01]	[1.02,1.03]	[1.00,1.01]	[1.00,1.00]
**BMI** (kg/m^2^)	0.98 ***	1.00	1.01 ***	1.00	1.01	0.99 ***	1.00	1.00 **	0.97 ***	0.99	0.99	1.00	0.97 ***	1.00	1.01 ***
	[0.97,0.99]	[1.00,1.00]	[1.00,1.02]	[0.99,1.00]	[1.00,1.02]	[0.98,0.99]	[1.00,1.00]	[1.00,1.00]	[0.97,0.98]	[0.97,1.01]	[0.97,1.00]	[0.99,1.00]	[0.96,0.98]	[1.00,1.01]	[1.01,1.02]
**Education**															
Low level	Ref.	Ref.	Ref.	Ref.	Ref.	Ref.	Ref.	Ref.	Ref.	Ref.	Ref.	Ref.	Ref.	Ref.	Ref.
High level	1.13 *	1.01 *	1.01	1.09 ***	1.06	1.02	1.01	1.05 ***	1.21 ***	1.51 ***	1.31 ***	0.99	1.05	0.87 ***	1.01
	[1.01,1.26]	[1.00,1.01]	[0.95,1.07]	[1.05,1.13]	[0.94,1.19]	[0.99,1.05]	[1.00,1.01]	[1.03,1.07]	[1.14,1.29]	[1.23,1.84]	[1.14,1.51]	[0.95,1.03]	[0.96,1.15]	[0.84,0.90]	[0.96,1.07]
**Work**															
Non-worker	Ref.	Ref.	Ref.	Ref.	Ref.	Ref.	Ref.	Ref.	Ref.	Ref.	Ref.	Ref.	Ref.	Ref.	Ref.
Worker	0.88	1.02 ***	1.05	0.90 ***	0.79 **	0.99	1.01	0.97 *	1.06	0.85	1.03	0.84 ***	1.07	0.96 *	0.95
	[0.77,1.00]	[1.01,1.03]	[0.97,1.13]	[0.86,0.94]	[0.68,0.92]	[0.95,1.03]	[1.00,1.01]	[0.95,1.00]	[0.98,1.14]	[0.66,1.09]	[0.86,1.23]	[0.80,0.87]	[0.95,1.20]	[0.92,1.00]	[0.89,1.01]
**Marital status**															
Single	Ref.	Ref.	Ref.	Ref.	Ref.	Ref.	Ref.	Ref.	Ref.	Ref.	Ref.	Ref.	Ref.	Ref.	Ref.
Married	1.15 *	1.00	1.11 **	1.04 *	1.00	0.95 ***	0.99	1.03 **	1.02	0.79 *	0.84 *	1.02	0.91	1.01	1.21 ***
	[1.03,1.28]	[1.00,1.01]	[1.04,1.18]	[1.00,1.09]	[0.89,1.12]	[0.92,0.98]	[0.99,1.00]	[1.01,1.05]	[0.95,1.09]	[0.65,0.97]	[0.73,0.96]	[0.98,1.07]	[0.83,1.00]	[0.98,1.05]	[1.15,1.28]
**Smoking**															
Non-smoker	Ref.	Ref.	Ref.	Ref.	Ref.	Ref.	Ref.	Ref.	Ref.	Ref.	Ref.	Ref.	Ref.	Ref.	Ref.
Smoker	1.03	1.00	1.13 **	0.96	0.76 ***	0.97	1.00	0.98	1.44 ***	0.95	0.89	1.09 ***	0.94	0.92 ***	1.02
	[0.90,1.19]	[0.99,1.01]	[1.05,1.21]	[0.91,1.01]	[0.65,0.89]	[0.93,1.00]	[0.99,1.01]	[0.96,1.00]	[1.33,1.55]	[0.74,1.22]	[0.74,1.06]	[1.04,1.14]	[0.84,1.06]	[0.88,0.96]	[0.96,1.09]
Ex-smoker	1.22 **	1.01 *	1.16 ***	1.02	0.84 **	1.01	1.00	1.01	1.39 ***	0.90	1.12	1.10 ***	1.18 **	0.94 **	1.03
	[1.08,1.38]	[1.00,1.02]	[1.08,1.24]	[0.98,1.07]	[0.73,0.96]	[0.98,1.05]	[1.00,1.01]	[0.98,1.03]	[1.29,1.49]	[0.70,1.14]	[0.95,1.32]	[1.05,1.15]	[1.06,1.31]	[0.90,0.98]	[0.97,1.09]
**Physical activity**															
No	Ref.	Ref.	Ref.	Ref.	Ref.	Ref.	Ref.	Ref.	Ref.	Ref.	Ref.	Ref.	Ref.	Ref.	Ref.
Yes	1.37 ***	1.00	0.92 **	1.18 ***	1.16 *	1.06 ***	1.01	1.06 ***	1.02	1.08	1.46 ***	1.10 ***	1.32 ***	1.09 ***	0.92 **
	[1.23,1.52]	[0.99,1.01]	[0.86,0.97]	[1.14,1.23]	[1.03,1.30]	[1.03,1.10]	[1.00,1.01]	[1.04,1.07]	[0.96,1.09]	[0.88,1.32]	[1.27,1.68]	[1.06,1.15]	[1.21,1.45]	[1.06,1.13]	[0.88,0.97]

Values are prevalence ratios (PR) with robust 95% confidence intervals obtained from a multivariable Poisson working regression model (PWRM). Abbreviations: sp = spoons; s = servings; u = units; gl = glass; t = times, Ref = reference category. * *p* < 0.05, ** *p* < 0.01, *** *p* < 0.001.

## Supplementary Materials

The following are available online at www.mdpi.com/2072-6643/9/7/734/s1, Figure S1: Marginal probabilities of adherence to the Mediterranean dietary pattern and its individual components as a function of age and nutritional status, Table S1: Estimated probabilities of adherence to the Mediterranean dietary pattern and individual components for each factor.
