# Supplementary material for: Trends of Adherence to the Mediterranean Dietary Pattern in Northern Italy from 2010 to 2016
Source: Nutrients. 2017 Jul 11;9(7):734. doi: 10.3390/nu9070734 (PMC5537848; doi:10.3390/nu9070734)
Supplement: Supplementary file 1 [file nutrients-09-00734-s001.pdf]

Table S1. Estimated probabilities of adherence to the Mediterranean dietary pattern and individual components for each factor.

	MDP (Yes)	Olive oil (Yes)	Olive oil (≥4 sp/d)	Vegetables (≥2 s/d)	Fruit (≥3 u/d)	Red meat (<1 s/d)	Animal fats (<1 s/d)	Sweetened beverages (<1 gl/d)	Wine (≥3 gl/w)	Legumes (≥3 s/w)	Fish (≥3 s/w)	Sweets (<3 t/w)	Nuts (≥1 s/w)	White meat (Yes)	Soffrito (≥2 t/w)
**Year**															
2010	0.12	0.97	0.27	0.53	0.16	0.66	0.97	0.82	0.28	0.05	0.10	0.52	0.09	0.57	0.56
	[0.11,0.14]	[0.96,0.98]	[0.25,0.30]	[0.51,0.56]	[0.14,0.18]	[0.64,0.69]	[0.96,0.98]	[0.80,0.84]	[0.26,0.30]	[0.04,0.06]	[0.09,0.12]	[0.49,0.55]	[0.08,0.11]	[0.54,0.60]	[0.54,0.59]
2011	0.15	0.97	0.37	0.57	0.14	0.67	0.97	0.84	0.31	0.05	0.08	0.55	0.12	0.57	0.54
	[0.13,0.17]	[0.97,0.98]	[0.35,0.40]	[0.54,0.60]	[0.12,0.16]	[0.65,0.70]	[0.96,0.98]	[0.82,0.86]	[0.28,0.33]	[0.04,0.06]	[0.07,0.10]	[0.52,0.57]	[0.10,0.14]	[0.55,0.60]	[0.51,0.56]
2012	0.15	0.97	0.41	0.51	0.12	0.70	0.98	0.83	0.33	0.04	0.08	0.57	0.15	0.58	0.37
	[0.13,0.16]	[0.96,0.98]	[0.38,0.44]	[0.48,0.54]	[0.10,0.14]	[0.67,0.72]	[0.97,0.99]	[0.81,0.85]	[0.31,0.36]	[0.03,0.05]	[0.06,0.09]	[0.54,0.60]	[0.13,0.17]	[0.56,0.61]	[0.34,0.40]
2013	0.13	0.97	0.39	0.52	0.13	0.69	0.98	0.84	0.31	0.03	0.09	0.55	0.17	0.62	0.32
	[0.11,0.15]	[0.96,0.98]	[0.37,0.42]	[0.50,0.55]	[0.11,0.14]	[0.67,0.72]	[0.97,0.99]	[0.82,0.86]	[0.28,0.34]	[0.02,0.05]	[0.07,0.10]	[0.52,0.57]	[0.15,0.19]	[0.60,0.65]	[0.29,0.34]
2014	0.14	0.98	0.33	0.56	0.10	0.67	0.98	0.85	0.31	0.05	0.09	0.57	0.20	0.63	0.33
	[0.12,0.16]	[0.97,0.98]	[0.30,0.35]	[0.54,0.59]	[0.08,0.12]	[0.64,0.69]	[0.97,0.99]	[0.83,0.87]	[0.29,0.34]	[0.04,0.06]	[0.07,0.10]	[0.55,0.60]	[0.18,0.22]	[0.60,0.65]	[0.31,0.36]
2015	0.15	0.97	0.33	0.57	0.10	0.70	0.98	0.87	0.31	0.04	0.08	0.57	0.24	0.61	0.34
	[0.13,0.17]	[0.96,0.98]	[0.30,0.35]	[0.54,0.59]	[0.09,0.12]	[0.67,0.72]	[0.97,0.98]	[0.85,0.89]	[0.28,0.33]	[0.03,0.06]	[0.07,0.10]	[0.54,0.59]	[0.21,0.26]	[0.58,0.64]	[0.31,0.37]
2016	0.14	0.98	0.32	0.56	0.09	0.72	0.98	0.87	0.30	0.05	0.09	0.56	0.26	0.62	0.34
	[0.12,0.16]	[0.97,0.99]	[0.30,0.35]	[0.53,0.58]	[0.08,0.11]	[0.70,0.75]	[0.97,0.99]	[0.86,0.89]	[0.28,0.33]	[0.04,0.06]	[0.08,0.11]	[0.53,0.59]	[0.23,0.28]	[0.60,0.65]	[0.31,0.37]
**Sex**															
Female	0.14	0.98	0.36	0.57	0.12	0.69	0.98	0.86	0.24	0.04	0.09	0.55	0.18	0.63	0.39
	[0.13,0.15]	[0.97,0.98]	[0.35,0.38]	[0.56,0.59]	[0.12,0.13]	[0.68,0.70]	[0.97,0.98]	[0.85,0.87]	[0.23,0.25]	[0.03,0.04]	[0.09,0.10]	[0.54,0.56]	[0.17,0.19]	[0.62,0.64]	[0.37,0.40]
Male	0.14	0.97	0.30	0.48	0.11	0.68	0.97	0.82	0.46	0.06	0.08	0.57	0.17	0.53	0.43
	[0.12,0.15]	[0.96,0.98]	[0.28,0.32]	[0.46,0.50]	[0.10,0.12]	[0.66,0.70]	[0.97,0.98]	[0.80,0.83]	[0.44,0.48]	[0.05,0.07]	[0.07,0.09]	[0.55,0.59]	[0.16,0.19]	[0.51,0.55]	[0.41,0.45]
**Education**															
Low level	0.13	0.97	0.35	0.53	0.12	0.68	0.97	0.83	0.28	0.04	0.08	0.56	0.17	0.64	0.40
	[0.12,0.14]	[0.97,0.98]	[0.33,0.36]	[0.51,0.54]	[0.11,0.13]	[0.67,0.70]	[0.97,0.98]	[0.82,0.84]	[0.27,0.29]	[0.03,0.04]	[0.07,0.09]	[0.54,0.57]	[0.16,0.18]	[0.62,0.65]	[0.39,0.41]
High level	0.15	0.98	0.35	0.57	0.12	0.69	0.98	0.87	0.34	0.06	0.10	0.55	0.18	0.55	0.40
	[0.14,0.16]	[0.97,0.98]	[0.33,0.36]	[0.56,0.59]	[0.11,0.14]	[0.68,0.71]	[0.98,0.98]	[0.86,0.88]	[0.33,0.36]	[0.05,0.07]	[0.09,0.11]	[0.54,0.57]	[0.17,0.19]	[0.53,0.57]	[0.39,0.42]
**Work**															

Non-worker	0.15	0.96	0.33	0.59	0.14	0.69	0.97	0.86	0.29	0.05	0.09	0.64	0.17	0.62	0.42
	[0.14,0.17]	[0.95,0.97]	[0.31,0.36]	[0.57,0.62]	[0.12,0.16]	[0.67,0.71]	[0.96,0.98]	[0.85,0.88]	[0.27,0.32]	[0.04,0.06]	[0.07,0.10]	[0.61,0.66]	[0.15,0.18]	[0.60,0.64]	[0.39,0.44]
Worker	0.13	0.98	0.35	0.53	0.11	0.69	0.98	0.84	0.31	0.04	0.09	0.53	0.18	0.59	0.40
	[0.13,0.14]	[0.97,0.98]	[0.34,0.36]	[0.52,0.55]	[0.10,0.12]	[0.67,0.70]	[0.97,0.98]	[0.83,0.85]	[0.30,0.32]	[0.04,0.05]	[0.08,0.10]	[0.52,0.54]	[0.17,0.19]	[0.58,0.61]	[0.38,0.41]
**Marital status**															
Single	0.13	0.97	0.33	0.53	0.12	0.71	0.98	0.83	0.30	0.05	0.10	0.55	0.19	0.60	0.36
	[0.12,0.14]	[0.97,0.98]	[0.31,0.34]	[0.52,0.55]	[0.11,0.13]	[0.69,0.72]	[0.98,0.98]	[0.82,0.85]	[0.29,0.32]	[0.04,0.06]	[0.09,0.11]	[0.53,0.56]	[0.17,0.20]	[0.58,0.61]	[0.34,0.37]
Married	0.15	0.98	0.36	0.56	0.12	0.67	0.97	0.86	0.31	0.04	0.08	0.56	0.17	0.60	0.44
	[0.14,0.16]	[0.97,0.98]	[0.35,0.38]	[0.54,0.57]	[0.11,0.13]	[0.66,0.68]	[0.97,0.98]	[0.85,0.87]	[0.30,0.32]	[0.04,0.05]	[0.07,0.09]	[0.55,0.57]	[0.16,0.18]	[0.59,0.62]	[0.42,0.45]
**Smoking**															
Non-smoker	0.13	0.97	0.33	0.55	0.13	0.69	0.98	0.85	0.26	0.05	0.09	0.53	0.17	0.62	0.40
	[0.12,0.14]	[0.97,0.98]	[0.31,0.34]	[0.53,0.56]	[0.12,0.14]	[0.68,0.70]	[0.97,0.98]	[0.84,0.86]	[0.24,0.27]	[0.04,0.05]	[0.08,0.10]	[0.52,0.55]	[0.16,0.18]	[0.61,0.64]	[0.38,0.41]
Smoker	0.14	0.97	0.37	0.53	0.10	0.67	0.98	0.83	0.37	0.05	0.08	0.58	0.16	0.57	0.41
	[0.12,0.15]	[0.97,0.98]	[0.34,0.39]	[0.50,0.55]	[0.09,0.11]	[0.65,0.69]	[0.97,0.98]	[0.81,0.85]	[0.35,0.39]	[0.04,0.05]	[0.07,0.09]	[0.55,0.60]	[0.15,0.18]	[0.55,0.59]	[0.38,0.43]
Ex-smoker	0.16	0.98	0.38	0.56	0.11	0.70	0.98	0.85	0.36	0.04	0.10	0.58	0.20	0.58	0.41
	[0.15,0.18]	[0.97,0.99]	[0.35,0.40]	[0.54,0.58]	[0.10,0.12]	[0.68,0.72]	[0.97,0.99]	[0.84,0.87]	[0.34,0.38]	[0.03,0.05]	[0.09,0.11]	[0.56,0.61]	[0.18,0.22]	[0.56,0.60]	[0.39,0.43]
**Physical activity**															
No	0.12	0.97	0.36	0.51	0.11	0.67	0.97	0.83	0.30	0.04	0.07	0.53	0.15	0.58	0.41
	[0.11,0.13]	[0.97,0.98]	[0.35,0.37]	[0.49,0.52]	[0.10,0.12]	[0.66,0.68]	[0.97,0.98]	[0.82,0.84]	[0.29,0.32]	[0.04,0.05]	[0.07,0.08]	[0.52,0.54]	[0.14,0.16]	[0.56,0.59]	[0.40,0.43]
Yes	0.16	0.97	0.33	0.60	0.13	0.71	0.98	0.87	0.31	0.05	0.11	0.59	0.20	0.63	0.38
	[0.15,0.18]	[0.97,0.98]	[0.31,0.34]	[0.58,0.61]	[0.12,0.14]	[0.70,0.73]	[0.98,0.98]	[0.86,0.88]	[0.30,0.33]	[0.04,0.05]	[0.10,0.12]	[0.57,0.60]	[0.19,0.22]	[0.62,0.65]	[0.37,0.40]

Values are marginal probabilities with 95% confidence intervals estimated for year of recruitment, sex, education, occupation, marital status, smoking status and physically activity level using the multivariable PWRM. Abbreviations: sp = spoons; d = day; s = servings; u=units; gl = glass; w = week; t = times.

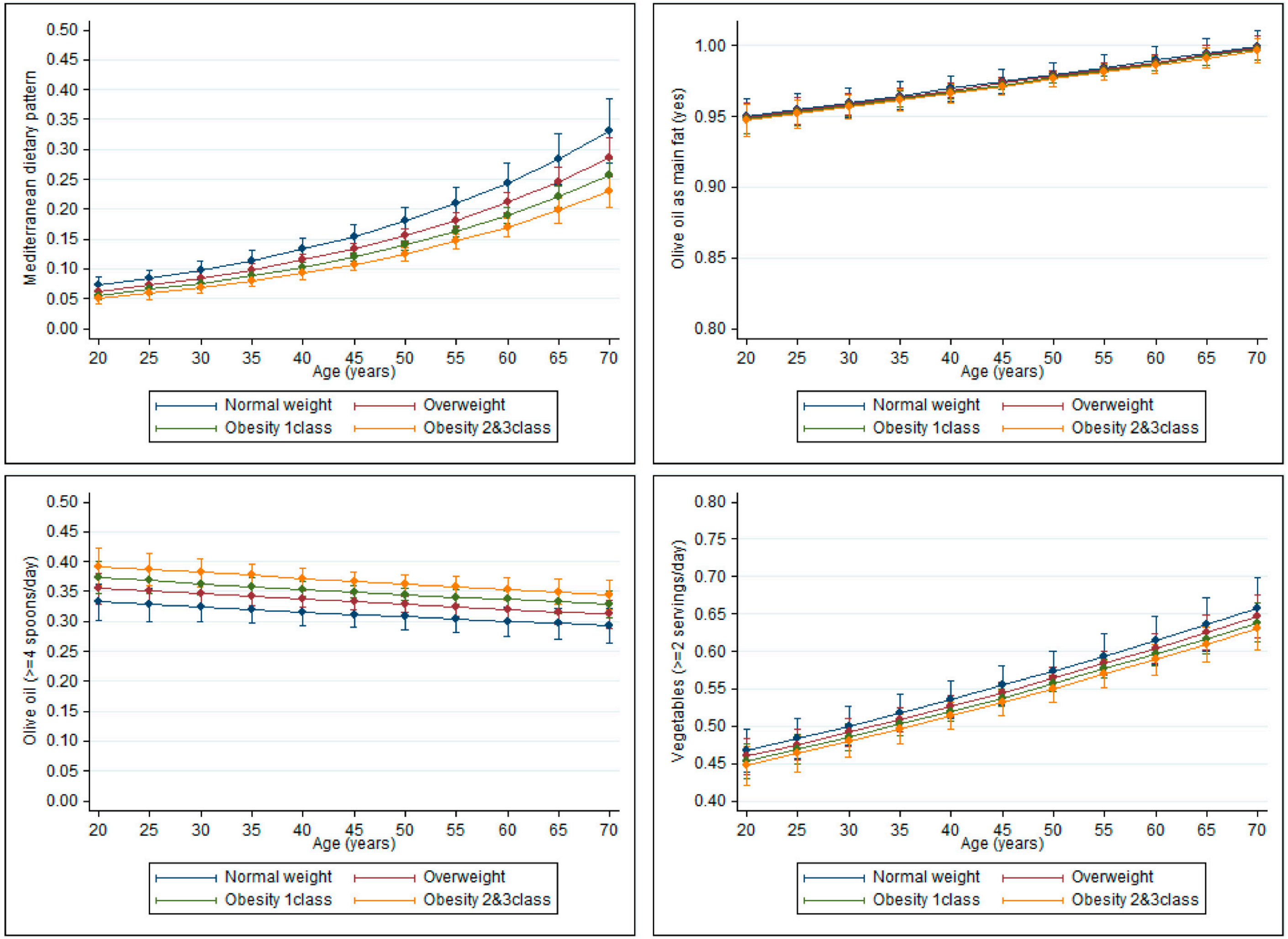
Mediterranean dietary pattern
Olive oil as main fat (yes)
Olive oil (>=4 spoons/day)
Vegetables (>=2 servings/day)
Age (years)
Normal weight
Overweight
Obesity 1class
Obesity 2&3class

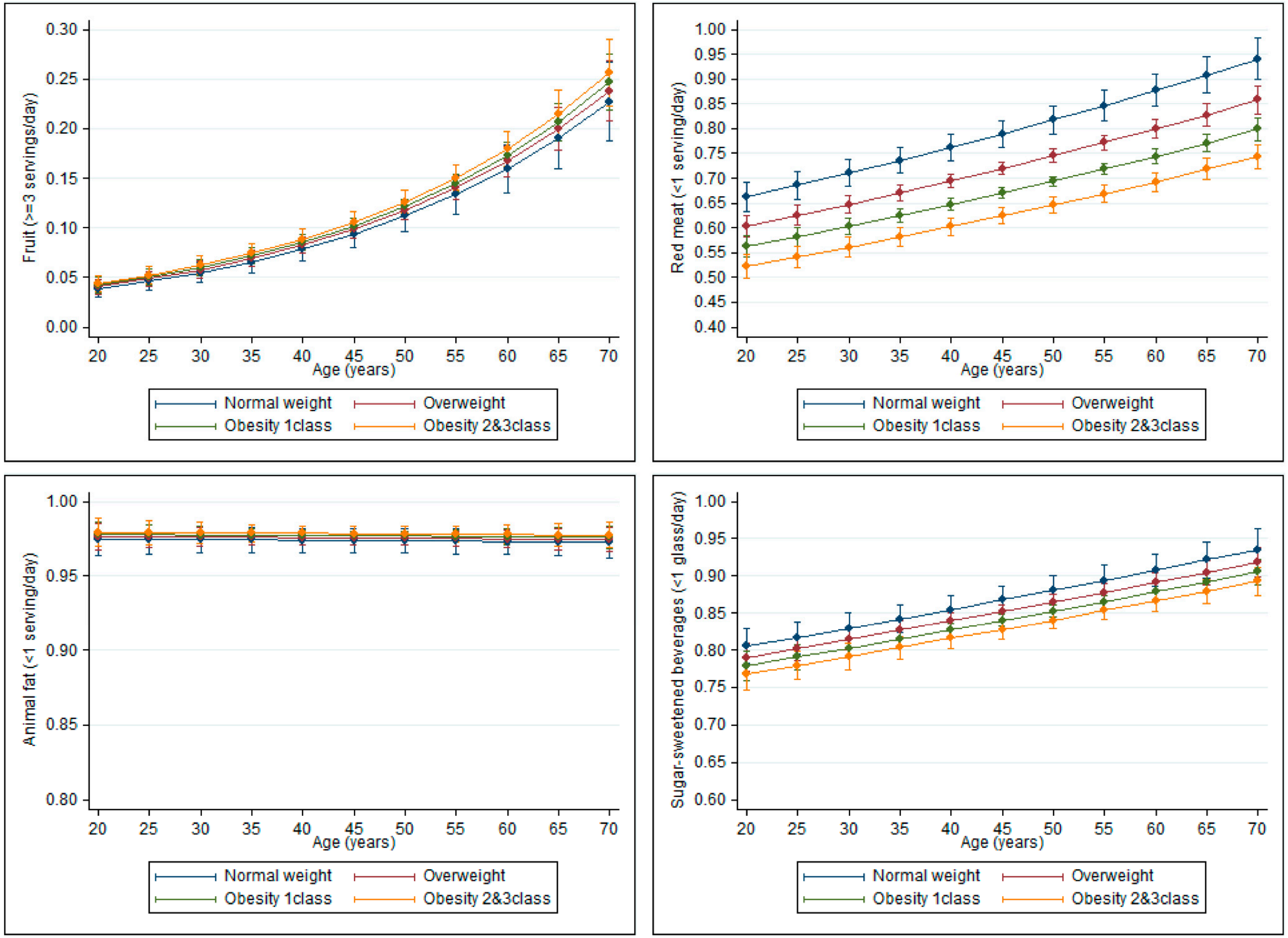
Fruit (>=3 servings/day)
0.00
0.05
0.10
0.15
0.20
0.25
0.30
Red meat (<1 serving/day)
0.40
0.45
0.50
0.55
0.60
0.65
0.70
0.75
0.80
0.85
0.90
0.95
1.00
Animal fat (<1 serving/day)
0.80
0.85
0.90
0.95
1.00
Sugar-sweetened beverages (<1 glass/day)
0.60
0.65
0.70
0.75
0.80
0.85
0.90
0.95
1.00
20 25 30 35 40 45 50 55 60 65 70
Age (years)
Normal weight
Overweight
Obesity 1class
Obesity 2&3class

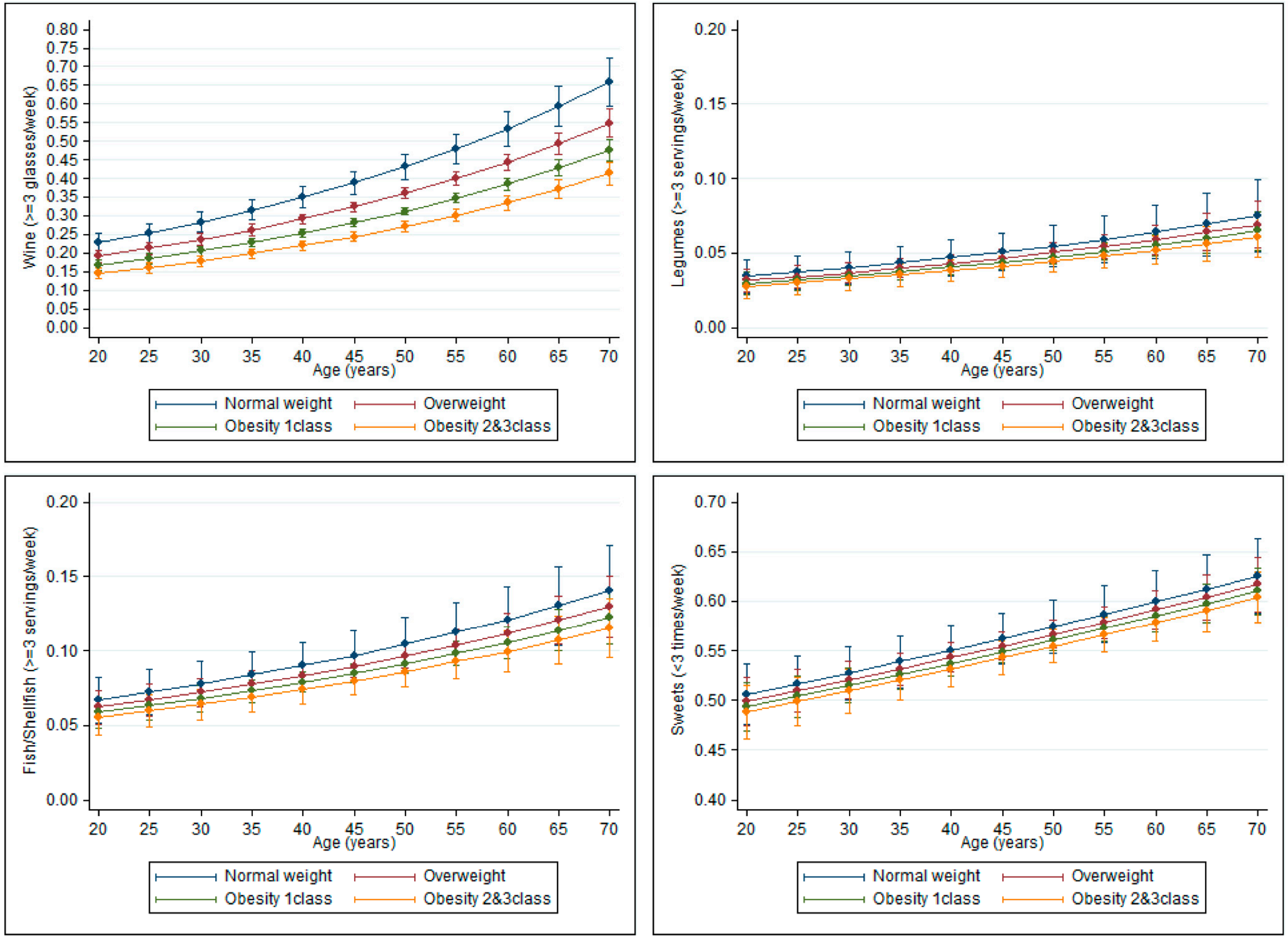
Wine (>=3 glasses/week)
0.80
0.75
0.70
0.65
0.60
0.55
0.50
0.45
0.40
0.35
0.30
0.25
0.20
0.15
0.10
0.05
0.00
20 25 30 35 40 45 50 55 60 65 70
Age (years)
Normal weight
Overweight
Obesity 1class
Obesity 2&3class
Legumes (>=3 servings/week)
0.20
0.15
0.10
0.05
0.00
20 25 30 35 40 45 50 55 60 65 70
Age (years)
Normal weight
Overweight
Obesity 1class
Obesity 2&3class
Fish/Shellfish (>=3 servings/week)
0.20
0.15
0.10
0.05
0.00
20 25 30 35 40 45 50 55 60 65 70
Age (years)
Normal weight
Overweight
Obesity 1class
Obesity 2&3class
Sweets (<3 times/week)
0.70
0.65
0.60
0.55
0.50
0.45
0.40
20 25 30 35 40 45 50 55 60 65 70
Age (years)
Normal weight
Overweight
Obesity 1class
Obesity 2&3class

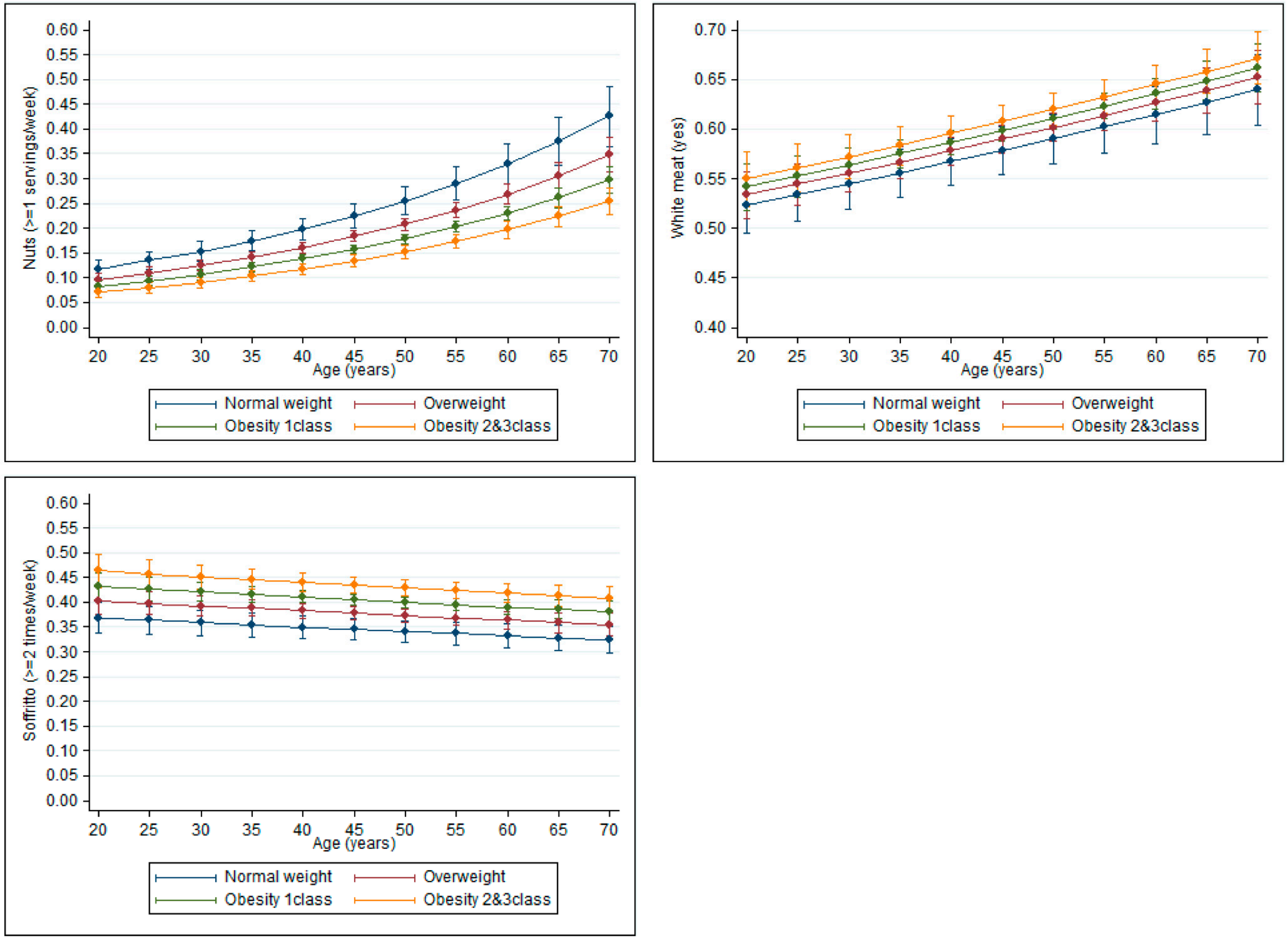

Figure S1. Marginal probabilities of adherence to the Mediterranean dietary pattern and its individual components as a function of age and nutritional status. The estimated probabilities were obtained using Poisson working regression models adjusted for socio-demographic characteristics, nutritional status, lifestyle variables and year of recruitment (see statistical analysis section for details).
